# Treatment Strategy in Reconstructive Microsurgery

**DOI:** 10.14789/ejmj.JMJ24-0053-R

**Published:** 2025-03-12

**Authors:** KAZUFUMI SANO

**Affiliations:** 1 Department of Plastic & Reconstructive Surgery, Juntendo University School of Medicine, Tokyo, Japan; 1 Department of Plastic & Reconstructive Surgery, Juntendo University School of Medicine, Tokyo, Japan

**Keywords:** microsurgery, free flap, finger replantation

## Abstract

Half a century has passed since microsurgical techniques were developed. These special techniques were exactly breakthrough in a variety of difficult situations in the field of reconstructive surgery. At present, the field of reconstructive microsurgery is further progressed with the development of microsurgical equipment and microscopes and matured enough to be considered with strategic approach.

## What is microsurgery?

Microsurgery is a specialized surgical technique that involves the use of high-powered microscopes and precision instruments to repair or reconstruct damaged or missing tissues, especially blood vessels and nerves. As a particular application, lymphatic anastomosis needs microsurgical technique as well. In recent years, digital microscope projecting a high-definition image of the operative field onto a three-dimensional monitor has been developed and allows surgeons to perform 3D-monitor-assisted microsurgery with heads-up style^1)^. There are two advantages compared to conventional optical microscopes. First, digital microscopes offer optimal ergonomics for surgeons to prevent fatigue and damage to their neck. Second, real digital image can be shared in all people in the operation room.

## Vascular anastomosis & neurorrhaphy

Intended external vascular diameters for microsurgery range from 0.3 to 3 mm. Proper size of suture thread is selected depending on the external diameter of vessels. Anastomosis using 11-0 or 12-0 is so called super-microsurgery. The necessary number of stiches ranges from six to ten, depending on vascular size.

In neurorrhaphy, namely nerve suture, epineural- perineural suture technique, in which each funiculus is approximated with single suture after penetrating the epineurium, is commonly employed with 9-0 or 10-0 nylon. Each funiculus usually needs 2 stitches. Unlike vascular suture using vascular clamps, nerves must hold gently while suturing.

## What is reconstructive microsurgery?

Microsurgery is an essential technique in finger replantation and revascularization, also used in tissue reconstruction. Reconstructive microsurgery means treatment of complex wounds, restoration of function, and improvement of appearance following trauma, cancer surgery, or congenital abnormalities using microsurgical techniques. Reconstructive microsurgery is synonymous with free flap surgery that involves the transfer of a patient’s own tissue from a donor site to a recipient site. The donor site usually has a distant location with respect to the recipient site. Therefore, in order to transfer tissue while maintaining its viability, vascular supply of tissue must be divided at the donor site and then reconnected through the microvascular anastomosis at the recipient site.

## Type of free flaps

Free flaps are commonly described in relation to their tissue composition. The type of flap can be composed of skin, fascia, muscle, bone, nerve, cartilage, or their combination with a single blood supply. For example, skin flaps include skin and superficial fascia. Fasciocutaneous flaps include skin and investing in a layer of deep fascia. Myocutaneous flaps include muscle and skin, and so on. Since muscle flaps and myocutaenous flaps have been most valuable in various reconstructive cases, anatomical vasculature of the whole muscle was actively studied more than half a century ago. Mathes and Nahai developed a classification system recognizing five basic patterns of muscle circulation^2)^. In type I muscles, such as the tensor fascia lata or the gastrocnemius, there is a single dominant vascular pedicle. Type II muscles like the gracilis have a dominant pedicle and minor or segmental pedicles. Type III muscles like the rectus abdominis or the gluteus maximus have two dominant pedicles, only one of which is necessary to supply the whole muscle. Type IV muscles like the sartorius or the tibialis anterior have a segmental blood supply with no dominant pedicle. Finally, type V muscles like the pectoralis major or the latissimus dorsi muscle, have a dominant pedicle and secondary segmental pedicles. In contrast to type II muscles, type V muscles can be supplied by secondary pedicles if the dominant pedicle gets sacrificed. Muscle flaps can be used locally, remaining attached to their blood supply in a pedicled fashion, or used for a distant reconstruction as a free tissue transfer, requiring microvascular anastomosis. From the beginning of this century, smaller and more superficial vasculatures have been revealed. Free flaps based on these fine vessels, so called perforator flaps, have been harvested from the whole body without sacrifice of the major vessels.

## Site-specific frequency of usable free flaps

### For calvarial reconstruction

The rectus abdominis and the latissimus dorsi muscle/myocutaneous flaps have broad utility for both tissue augmentation and skin coverage in this region. Perforator flaps, especially anterolateral thigh (ALT) flaps, are most popular flap for coverage of the superficial defect. ALT flaps are nourished by the skin perforator of the descending branch of the lateral circumflex femoral vessels.

### For facial reconstruction

Before innovation of perforator flaps, the radial forearm fasciocutaneous flap used to be most popular because of its thin and elastic behavior. It is harvested with the radial artery and the concomitant veins. The cephalic vein can be also used for venous drainage root. The perforator flaps like ALT flaps do not sacrifice main artery, have been recently substituted for the conventional radial forearm flaps.

The jejunum is also essential material for head and neck reconstruction. 10 to 20 cm of jejunum can be harvested with the second intestinal branch of the superior mesenteric vessels for pharyngeal reconstruction.

### For chest reconstruction

In this field, the rectus abdominis muscle/myocutaneous flap and its modifications are most frequently used. Deep inferior epigastric perforator (DIEP) flaps are extremely useful for breast reconstruction with autogenous tissue.

### For reconstruction of upper extremities

In the upper limb region, reconstruction is emphasized on functional improvement. Therefore, free functional muscle transfer using the gracilis muscle/ myocutaneous flap is a popular procedure^3)^. For finger reconstruction, free toe transfer and its modifications are like nothing else. Otherwise, small sized skin flaps are preferred. For small wound coverage is often demanded in hand and forearm regions. A medial sural artery perforator (MSAP) flap is a good candidate for these situations^4)^. Nourishing vessels of the medial sural artery perforator flaps are musculocutaneous perforators originate from the medial sural artery. Most of these perforators are clustered in the distal half of the gastrocnemius muscle and arise near the raphe separating the medial and lateral heads. This flap is nearly the same thickness as the radial forearm flap but does not sacrifice major vessels.

### For reconstruction of lower extremities

A free osseous or osteocutanoeus flap is frequently used for bony reconstruction. For reconstruction in this region, flaps from the subscapular axis are very useful^5)^. Since the latissimus dorsi muscle, the serratus anterior muscle, the scapular & parascapular skin flaps, and the outer edge of the scapular bone are nourished by the same vascular trunk, these flaps can be taken together with one nourishing vascular pedicle and transferred all together for complex wound management.

## Funding

No funding was received.

## Author contributions

The author read and approved of the final manuscript.

## Conflicts of interest statement

The author declares that there are no conflicts of interest.

## References

[1] Ichikawa Y, Tobita M, Takahashi R, et al: Learning curve and ergonomics associated with the 3D-monitor-assisted microsurgery using a digital microscope. J Plast Reconstr Surg, 2023; 2: 1-8. 10.53045/jprs.2021-0026 PMC12078115 40386791

[2] Mathes SJ, Nahai F: Clinical applications for muscle and musculocutaneous flaps. St. Louis: CV Mosby, 1982.

[3] Spinner RJ, Shin AY, Elhassan BT, Bishop AT: Traumatic brachial plexus injury. In: Wolfe SW, Hotchikiss RN, Pederson WC, Kozin SH, Cohen MS, eds. Green’s operative hand surgery 7th ed. Philadelphia: Elsevier, 2017: 1146-1207.

[4] SanoK, Hallock GG, Rice DC: Sural artery perforator flap. In: Blondeel PN, Morris SF, Hallock GG, Neligan PC, eds. Perforator Flaps 2nd ed. St Louis: Quality Medical Publishing, 2013: 803-816.

[5] Sano K, Hallock GG, Ozeki S, et al: Devastating massive knee defect reconstruction using the cornucopian chimera flap from the subscapular axis: two case reports. J Reconstr Microsurg, 2006; 22: 25-32. 16425118 10.1055/s-2006-931903

